# Implication of Interleukin-12/15/18 and Ruxolitinib in the Phenotype, Proliferation, and Polyfunctionality of Human Cytokine-Preactivated Natural Killer Cells

**DOI:** 10.3389/fimmu.2018.00737

**Published:** 2018-04-16

**Authors:** Iñigo Terrén, Idoia Mikelez, Irati Odriozola, Andrea Gredilla, Javier González, Ane Orrantia, Joana Vitallé, Olatz Zenarruzabeitia, Francisco Borrego

**Affiliations:** ^1^Immunopathology Group, BioCruces Health Research Institute, Barakaldo, Spain; ^2^CIC biomaGUNE, Donostia-San Sebastián, Spain; ^3^Ikerbasque, Basque Foundation for Science, Bilbao, Spain; ^4^Basque Center for Transfusion and Human Tissues, Galdakao, Spain

**Keywords:** natural killer cells, ruxolitinib, memory-like natural killer cells, cytokine preactivation, cytokine production, polyfunctionality, degranulation

## Abstract

A brief *in vitro* stimulation of natural killer (NK) cells with interleukin (IL)-12, IL-15, and IL-18 endow them a memory-like behavior, characterized by higher effector responses when they are restimulated after a resting period of time. These preactivated NK cells, also known as cytokine-induced memory-like (CIML) NK cells, have several properties that make them a promising tool in cancer immunotherapy. In the present study, we have described the effect that different combinations of IL-12, IL-15, and IL-18 have on the generation of human CIML NK cells. Our data points to a major contribution of IL-15 to CIML NK cell-mediated cytotoxicity against target cells. However, the synergistic effect of the three cytokines grant them the best polyfunctional profile, that is, cells that simultaneously degranulate (CD107a) and produce multiple cytokines and chemokines such as interferon γ, tumor necrosis factor α, and C-C motif chemokine ligand 3. We have also analyzed the involvement of each cytokine and their combinations in the expression of homing receptors CXCR4 and CD62L, as well as the expression of CD25 and IL-2-induced proliferation. Furthermore, we have tested the effects of the Jak1/2 inhibitor ruxolitinib in the generation of CIML NK cells. We found that ruxolitinib-treated CIML NK cells expressed lower levels of CD25 than non-treated CIML NK cells, but exhibited similar proliferation in response to IL-2. In addition, we have also found that ruxolitinib-treated NK cells displayed reduced effector functions after the preactivation, which can be recovered after a 4 days expansion phase in the presence of low doses of IL-2. Altogether, our results describe the impact that each cytokine and the Jak1/2 pathway have in the phenotype, IL-2-induced proliferation, and effector functions of human CIML NK cells.

## Introduction

Natural killer (NK) cells are innate lymphocytes with a crucial role in the defense against transformed cells and viral infections. They perform their work through different processes such as cell-mediated cytotoxicity and secretion of cytokines and chemokines including interferon (IFN)γ, tumor necrosis factor (TNF)α, and C-C motif chemokine ligand (CCL)3 (1–4). Human NK cells are defined by the expression of CD56 (natural cell adhesion molecule) and the lack of CD3/TCR (T cell receptor) complex (1, 3). The activation of NK cells is controlled by a balance between signals from inhibitory and activating receptors (5, 6). Among the firsts, there are major histocompatibility complex (MHC) class I-specific receptors such as the highly polymorphic inhibitory killer immunoglobulin-like receptors (KIRs) that bind to MHC class Ia ligands (HLA-A, -B, and -C), and the inhibitory CD94/NKG2A heterodimeric receptor that binds to MHC class Ib ligands (HLA-E). In addition, NK cells express other inhibitory receptors such as CD300a, TIGIT, LAIR-1, etc. (4, 7, 8). On the other side, NK cells express a wide repertoire of activating receptors, including the natural cytotoxicity receptors (NKp30, NKp44, NKp46), C-type lectin-like receptors (CD94/NKG2C, CD94/NKG2E, and the homodimer NKG2D), activating KIRs, the antibody-binding receptor CD16 (or FcγRIIIa), DNAM-1, 2B4, NKp80, CD300c, etc. (1, 4, 5, 9, 10).

Although NK cells have been traditionally classified as part of the innate immune system, recent publications have shown that they exhibit several immunological memory features. The first evidence of the existence of memory NK cells was described in mice lacking T and B cells. They developed long lasting and specific contact hypersensitivity responses to different haptens that were mediated by NK cells (11). Later, it was demonstrated that mouse NK cells also exhibited specific memory responses to mouse cytomegalovirus (12), vaccinia virus (13), and to vaccines containing antigens from influenza virus, vesicular stomatitis virus, or human immunodeficiency virus (HIV) (14). In non-human primates, it has been described antigen specific NK cell memory responses in chronically simian-human immunodeficiency virus and simian immunodeficiency virus-infected rhesus macaques, and also in animals vaccinated with replication-incompetent adenovirus vector (Ad26) expressing HIV-1 Env or DNA–Ad26 prime-boost vaccine expressing SIV_mac239_ Gag (15). In humans, infection by human cytomegalovirus (HCMV) promotes an adaptive expansion of long-lived NK cells that are characterized by the expression of the CD94/NKG2C receptor (16–18). These adaptive NKG2C+ NK cells exhibit higher effector functions than canonical NKG2C− NK cells and have been associated with the control of HCMV infection in kidney transplant recipients and with protection from leukemia relapse after allogeneic hematopoietic stem cell transplantation (HSCT) (17, 19–24). Furthermore, exposure of NK cells to a combination of interleukin (IL)-12, IL-15 plus IL-18 for a short period of time (16–18 h) results in a memory-like behavior in the absence of antigen. These cytokine-induced memory-like (CIML) NK cells are characterized by enhanced effector functions after a resting period of time (25–27). For example, human CIML NK cells exhibit greater IFNγ and TNFα production, increased expression of granzyme B and perforin, and superior cytotoxicity in response to tumor targets *in vitro* (27–31). Furthermore, in xenograft mouse models, CIML NK cells were found to be effective (26, 27).

Natural killer cells are of obvious clinical interest, and they are currently being explored as a potent tool for the treatment of hematological malignancies (32–34). One of the first clinical observations that demonstrated the relevant role of NK cells in the fight against cancer was the appreciation that in patients with acute myeloid leukemia (AML) that were subjected to an allogeneic HSCT, the success of it was greater when donor subsets of NK cells possessed a KIR repertoire that do not interact with HLA class I molecules in the recipient. In this way, these alloreactive NK cells can exert a graft-versus-leukemia effect in the absence of graft-versus-host disease (35). The ability of human NK cells to lyse leukemic cells *in vivo* in the context of patients who undergo HSCT, demonstrates that NK cells participate in the immunosurveillance against the development of leukemias. However, despite recent progress, there are still many patients with a poor prognosis. In these cases, adoptive NK cell therapy could offer a relatively safe and effective alternative. In fact, many clinical trials with NK cell infusions have been completed or are underway (34, 36–38). Interestingly, there are several ongoing clinical trials using CIML NK cells (27) (NCT01898793, NCT03068819, and NCT02782546).

In this work, we have studied the contribution of each cytokine (IL-12, IL-15, and IL-18) to the phenotype, proliferation, and polyfunctionality of human CIML NK cells. Furthermore, considering that IL-12 and IL-15 induced signals involve Jak2 and Tyk2 and Jak1/3 pathways, respectively, we decided to investigate the role of the specific Jak1/2 inhibitor ruxolitinib, a drug that has been approved for the treatment of myelofibrosis, in the generation of CIML NK cells. Altogether, our results may contribute to a better knowledge about human CIML NK cells and their properties.

## Materials and Methods

### Subjects and Samples

Blood samples from adult healthy donors were collected through the Basque Biobank for Research (http://www.biobancovasco.org). The Basque Biobank complies with the quality management, traceability, and biosecurity, set out in the Spanish Law 14/2007 of Biomedical Research and in the Royal Decree 1716/2011. All subjects provided written and signed informed consent in accordance with the Declaration of Helsinki. The protocol was approved by the Basque Ethics Committee for Clinical Research (PI + INC-BIOEF 2014-02 14-27 and PI2014079). As indicated in each figure legend, samples from 4 to 8 different donors were used for each set of experiments.

### Antibodies and Reagents

The following fluorochrome conjugated mouse anti-human mAbs were used for flow cytometric analysis: PE anti-CD25 (M-A251), APC-Cy7 anti-CD184/CXCR4 (12G5), PerCP-Cy5.5 anti-TNFα (MAb11), PerCP-Cy5.5 anti-CD3 (SK7), APC anti-TNFα (MAb11), APC anti-CD56 (MEM-188), PE anti-CD56 (MEM-188), BV510 anti-CD56 (5.1H11) from BioLegend; PE-Cy7 anti-CD3 (SK7), PE-Cy7 anti-CD184/CXCR4 (12G5), PE-Cy7 anti-IFNγ (B27), BV510 anti-IFNγ (B27), BV510 anti-CD62L (DREG-56), BV421 anti-CD107a (H4A3), PE anti-CD16 (3G8), Pacific Blue mouse anti-signal transducer and activator of transcription (STAT)5(pY694) (47/Stat5(pY694)) from BD Biosciences; and PE anti-CCL3 (MIP-1 alpha), APC-Cy7 anti-CD3 (SK7) from eBioscience. For flow cytometric analyses were also used LIVE/DEAD™ Fixable Near-IR Dead Cell Stain Kit (Invitrogen) and CellTrace™ CFSE Cell Proliferation Kit (Thermo Fisher Scientific).

The following reagents were also used: recombinant human IL-12 (Miltenyi Biotec), recombinant human IL-15 (Miltenyi Biotec), recombinant human IL-18 (MBL International), recombinant human IL-2 (Miltenyi Biotec), Jak1/2 inhibitor ruxolitinib (INCB018424, Selleckchem), BD GolgiStop™ Protein Transport Inhibitor (monensin), BD GolgiPlug™ Protein Transport Inhibitor (brefeldin A), BD Cytofix/Cytoperm™ Plus Kit (including Fixation/Permeabilization solution and BD Perm/Wash™ Buffer), BD Cytofix Fixation Buffer, BD Phosflow™ Perm Buffer III, Brilliant Buffer (BD Biosciences), paraformaldehyde (Sigma-Aldrich), calcein AM (Life Technologies), and Triton™ X-100 (Sigma-Aldrich).

### Cell Isolation and Cell Culture

**Figure 1:**
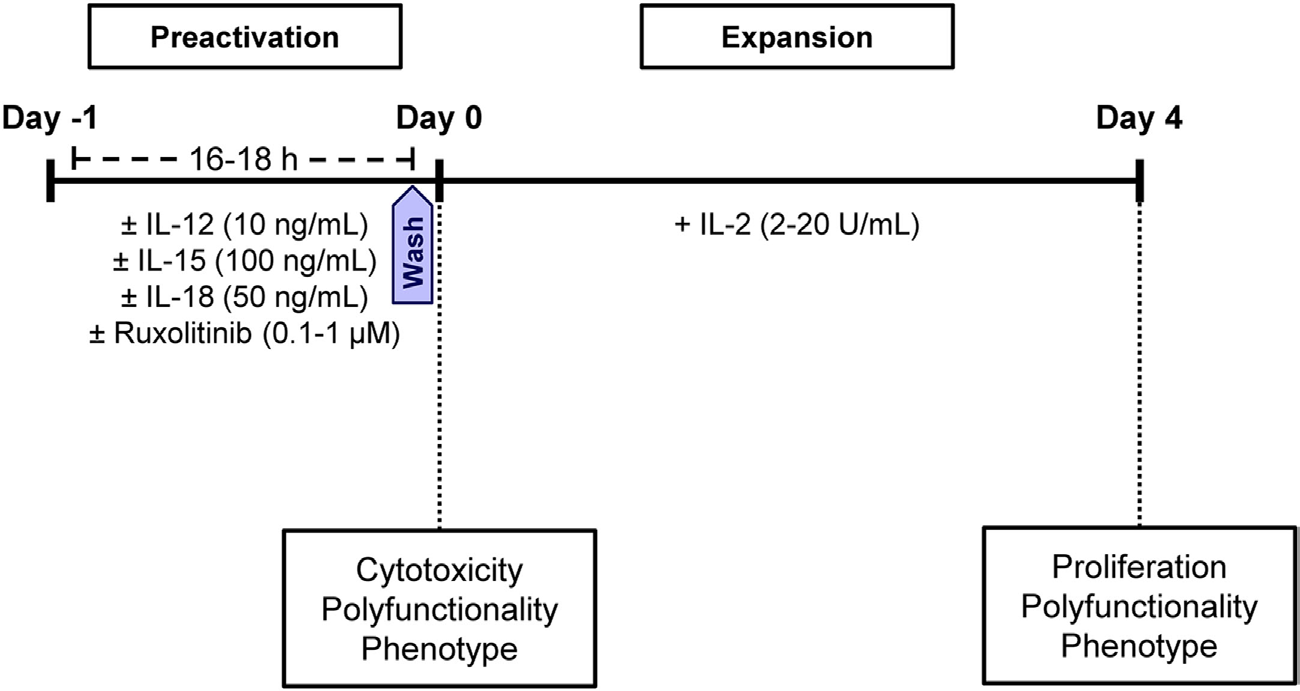
Graphic representation showing the culture conditions of natural killer (NK) cells. During the preactivation phase (16 –18 h), cells were stimulated with different combinations of IL-12, IL-15, and IL-18 in the presence and absence of ruxolitinib. During the expansion phase, NK cells were cultured with IL-2 for 4 days. At the end of the preactivation and expansion phases, the phenotype and effector functions of NK cells were tested.

Fresh peripheral blood mononuclear cells (PBMCs) were obtained from buffy coats by ficoll (GE Healthcare) density centrifugation. Isolated PBMCs were cultured at 37°C in Iscove’s Modified Dulbecco’s Medium (IMDM) (Thermo Fisher Scientific) supplemented with 10% human AB serum (Invitrogen), 1% GlutaMax (Thermo Fisher Scientific), and 1% penicillin/streptavidin (Thermo Fisher Scientific), hereinafter, complete IMDM or cIMDM. PBMCs were plated at 2 × 10^6^ cell/mL in 24-well plates and cultured at 37°C in cIMDM for 16–18 h (preactivation phase) with IL-12, IL-15, and/or IL-18 (10, 100, and 50 ng/mL, respectively) and in the presence and absence of 0.1–1 µM ruxolitinib. Next, cells were washed three times with phosphate-buffered saline (PBS) and plated at 2 × 10^6^ cell/mL in new 24-well plates, and cultured at 37°C in cIMDM for 4 days (expansion phase) with 2–20 U/mL IL-2 (Figure 1).

### Calcein-AM-Based Cytotoxicity Assay

This assay was performed following a previous published protocol (39). After the preactivation phase, PBMCs were washed twice with PBS. The target cell line K562 (10^6^ cells/mL) was incubated in the presence of 15 µM of calcein-AM for 30 min at 37°C in cRPMI (RPMI medium plus 10% fetal bovine serum, 1% penicillin/streptavidin, 1% GlutaMax, 1% non-essential aminoacids, and 1% sodium pyruvate). Then, target cells were washed twice. Calcein-AM labeled K562 cells (5,000 cells per well) were cocultured with PBMCs in 96 *U*-bottom well plates for 3 h at 37°C at 10:1 and 5:1 effector:target (E:T) ratios. For the measurement of spontaneous release, K562 target cells were incubated with no PBMCs. Total released was achieved by adding 2% Triton™ X-100 (Sigma-Aldrich) to the target cells. Triplicates were performed for all the conditions. After the incubation, 75 µL of the supernatant were harvested and transferred to a black 96-well plate to measure calcein-AM release in a Fluoroskan Ascent (Thermo Fisher Scientific), with the excitation and the bandpass filters adjusted to 485 ± 9 and 538 ± 9 nm, respectively. The percentage of specific lysis was calculated with the following formula: 100 × ([(Test release) − (Medium fluorescence)] − [(Spontaneous release) − (Medium fluorescence)])/([(Maximum release) − (Triton fluorescence)] − [(Spontaneous release) − (Medium fluorescence)]).

### Flow Cytometry Analysis

For proliferation assays, 4 × 10^6^ PBMCs/mL were labeled with 0.5 µM CFSE before the preactivation phase following manufacturer’s recommendations. For polyfunctionality analysis after the preactivation phase, PBMCs were incubated for 6 h at 37°C with Golgi Stop, Golgi Plug, and fluorochrome conjugated anti-CD107a mAb. For polyfunctionality analysis after the expansion phase, PBMCs were also incubated in the presence and absence of K562 cells during 6 h. Cells were washed twice with staining buffer containing PBS supplemented with 2.5% of bovine serum albumin, and stained for cell surface markers with the respective fluorochrome conjugated mAbs for 30 min on ice protected from light. For intracellular staining, cells were fixed and permeabilized with BD Cytofix/Cytoperm™ Plus Kit following the manufacturer’s protocol. For analysis after the expansion phase (4 days in the presence of IL-2), cells were also stained with LIVE/DEAD™ kit to exclude dead cells. Finally, cells were washed twice and acquired in a FACSCanto II Flow Cytometer (BD Biosciences). NK cells were gated as CD3−CD56+. Flow cytometry data were analyzed with FlowJo v7.6.5 and v10.0.7 (TreeStar).

For phosphorylation assays, freshly isolated PBMCs were washed with PBS and plated into 96 well plates at 10^6^ cell/mL in cIMDM. Then, cells were stimulated, in the presence and absence of 0.1 µM ruxolitinib, with cytokines [IL-12 (10 ng/mL), IL-15 (10 ng/mL), and IL-18 (50 ng/mL)] for 20 min at 37°C while they were stained for the surface markers CD3 and CD56. After the incubation, cells were harvested and washed with PBS before fixing them with the BD Cytofix buffer at 37°C for 10 min. Cells were washed again two times and permeabilized with the BD Phosflow Perm buffer III for 30 min on ice. Next, they were incubated for 30 min on ice with specific labeled phospho-STAT5 (pSTAT5) Abs. Finally, cells were washed to remove unbound Abs and further acquired in a FACSCanto II Flow cytometer. Data were analyzed with FlowJo software.

### Statistical Analysis and Data Representation

GraphPad Prism v6.01 and SPICE v5.3 (Vaccine Research Center, NIAID) (40) softwares were used for graphical representation and statistical analysis. Data were represented showing means ± SEM. Prior to statistical analyses, data were tested for normal distribution with D’Agostino and Pearson normality test. If data were normally distributed, RM one-way ANOVA with the Greenhouse-Geisser correction was used to determine significant differences. Non-normal distributed data were compared with non-parametric Friedman test (for multiple comparisons) or non-parametric Wilcoxon matched-pairs signed rank test, as indicated in each figure legend. Differences between pie charts were determined with the non-parametric permutation test with 100,000 iterations. *p < 0.05, **p < 0.01, ***p < 0.001, ****p < 0.0001.

## Results

### Cytotoxic Activity of Cytokine-Preactivated NK Cells Against K562 Target Cells

As it has been previously reported, NK cells can be preactivated with different combinations of cytokines (41–43). CIML NK cells are generated by the preactivation for a short period of time with a cocktail of cytokines including IL-12, IL-15, and IL-18 (25, 28). *In vitro*, human CIML NK cells exhibit enhanced effector functions after a resting phase that could last for weeks (27, 28, 30, 44). *In vivo*, preclinical models have shown that IL-12/15/18-preactivated NK cells exhibit sustained effector functions against established tumors (45). Importantly, adoptively transferred CIML NK cells (after 12–16 h of preactivation) have shown a clinical benefit in the treatment of patients with AML (27).

**Figure 2:**
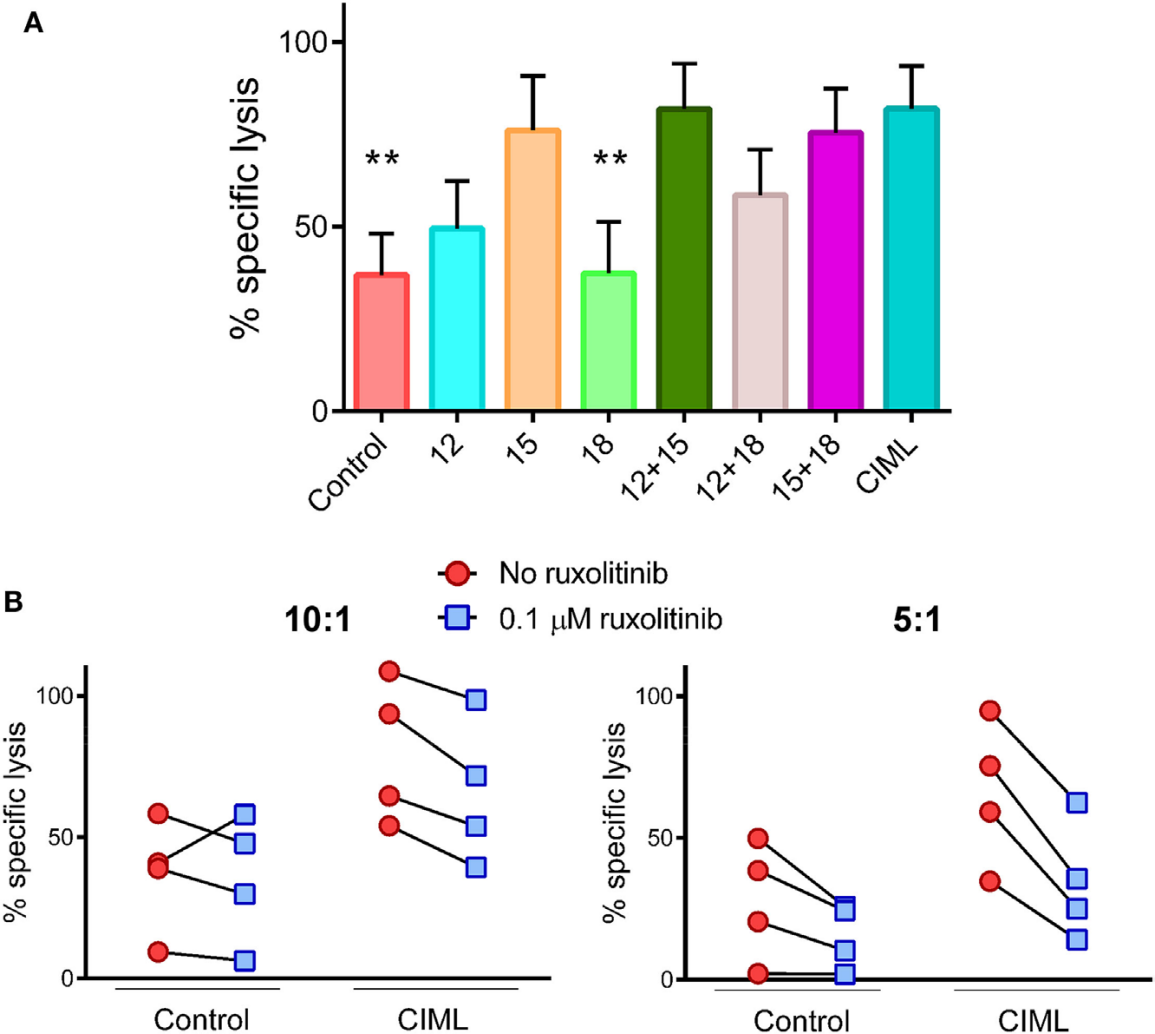
Cytotoxicity of cytokine preactivated natural killer (NK) cells at the end of the preactivation phase. **(A)** Bar graph showing the specific lysis of K562 target cells by control non-preactivated and cytokine preactivated NK cells at 10:1 E:T ratio (*n* = 4). The bar graph shows the mean with SEM. Differences between cytokine-induced memory-like (CIML) NK cells and the rest of conditions were established with Friedman test. **(B)** Dot plot graphs showing the specific lysis of K562 target cells by control non-preactivated and CIML NK cells exposed or not to 0.1 µM ruxolitinib at 10:1 (left) and 5:1 (right) E:T ratios (*n* = 4). **p < 0.01.

In order to study the contribution of each cytokine to the generation of CIML NK cells, we have analyzed the involvement of IL-12, IL-15, and IL-18 either alone or in different combinations. First, we tested the cytotoxic activity of human NK cells against K562 cancer cells, right after the preactivation phase. We demonstrated that cells stimulated for 16–18 h with IL-15 either alone or in combination with other cytokines exhibited the highest cytotoxic activity against K562 target cells (Figure 2A). Given that IL-15R acts through the Jak1/3 pathways and IL-12R involves signaling through Jak2 and Tyk2 pathways (46, 47), we investigated whether the presence of ruxolitinib (a specific Jak1/2 inhibitor) during the preactivation phase could affect the cytolytic potential of CIML NK cells. We first tested ruxolitinib-mediated inhibition by determining the phosphorylation of STAT5, which is involved in the IL-15R signaling pathway (48). Our results showed a strong phosphorylation of STAT5 in NK cells stimulated with IL-12, IL-15, and IL-18 in the absence of ruxolitinib. However, the levels of STAT5 phosphorylation in cytokine-stimulated NK cells in the presence of ruxolitinib were similar to non-stimulated NK cells (Figure S1 in Supplementary Material). Since signaling through IL-15R is partially impaired when Jak1 is inhibited, we predicted that ruxolitinib will reduce the killing ability of CIML NK cells. To prove our assumption, during the preactivation phase, PBMCs were exposed or not to ruxolitinib. As expected, CIML NK cell cytotoxic activity was diminished when they were generated in the presence of ruxolitinib (Figure 2B). We also observed that control non-preactivated NK cells exhibited a reduced cytotoxic activity, although not statistically significant, when they were incubated for 18 h with ruxolitinib.

### Polyfunctionality of Cytokine-Stimulated NK Cells

**Figure 3:**
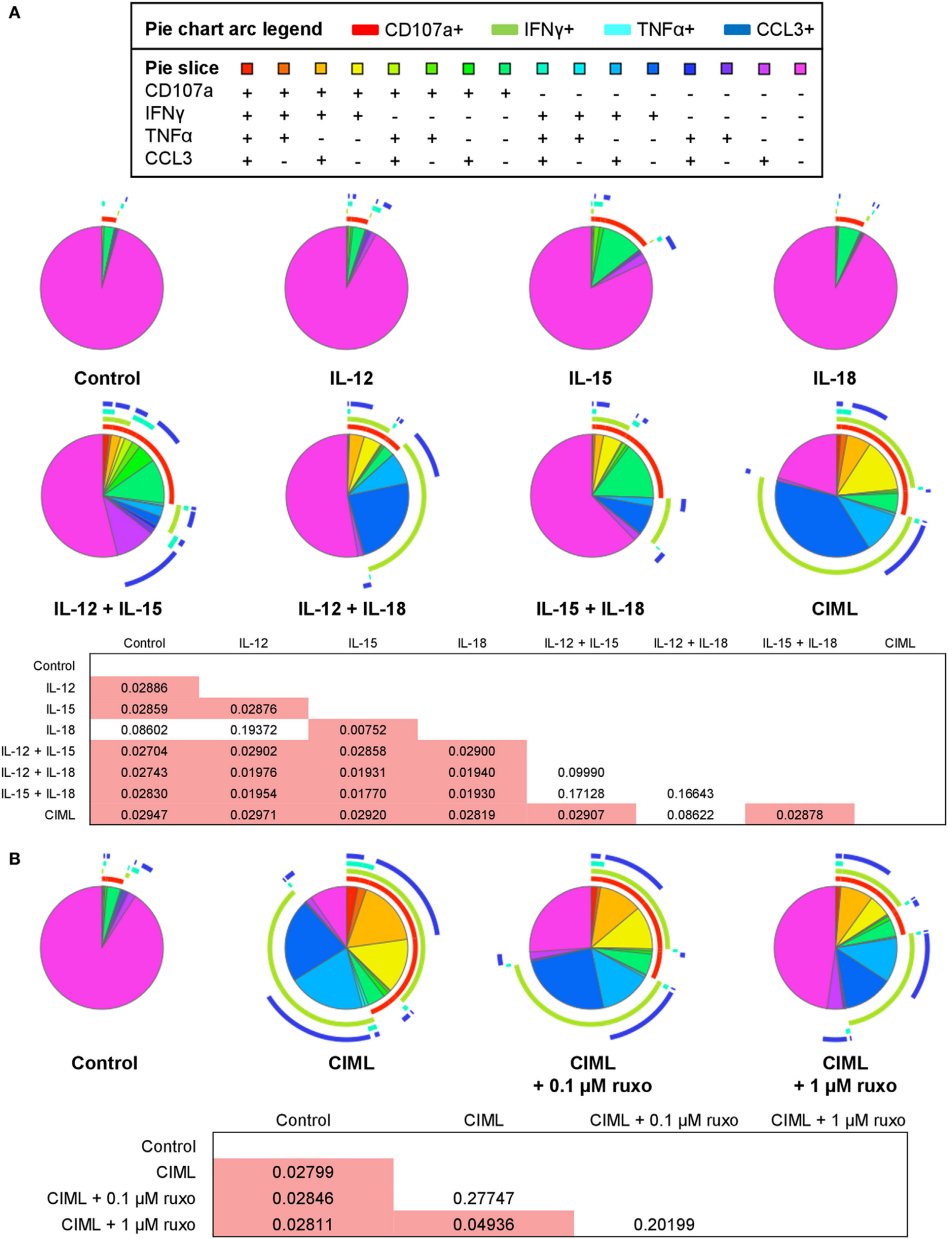
Polyfunctionality of cytokine preactivated natural killer (NK) cells after the preactivation phase. **(A)** Pie charts representing the percentages of control non-preactivated and cytokine preactivated NK cells expressing CD107a, interferon (IFN)γ, tumor necrosis factor (TNF)α, and/or C-C motif chemokine ligand (CCL)3 (*n* = 4). **(B)** Pie charts representing the percentages of control non-preactivated and cytokine-induced memory-like (CIML) NK cells, and CIML NK cells preactivated in the presence of 0.1 and 1 µM ruxolitinib, expressing CD107a, IFNγ, TNFα, and/or CCL3 (*n* = 4). Differences between pie charts were established with non-parametric permutation test. The *p*-values are in the boxes below the pie charts. Significant differences are in the red cells.

To further analyze the effector functions of cytokine-stimulated NK cells, we next examined the contribution of each cytokine to NK cell polyfunctionality, which is defined by those cells that simultaneously produce multiple cytokines and degranulate (49, 50). To determine polyfunctionality, we measured the expression of the extracellular CD107a as a marker of degranulation (51), and the production of IFNγ, TNFα, and CCL3. Our results revealed that CIML NK cells are significantly the most polyfunctional cells and that IL-12+IL-18 is the only combination of cytokines that generate NK cells with a similar polyfunctional profile (Figure 3A). Furthermore, the frequency of NK cells that are non-functional gradually decreases as more cytokines are used for the preactivation, being the combination of IL-12 + IL-15 + IL-18 (i.e., CIML NK cells) the one with the lower percentage of non-functional cells (Figure 3A). These results indicate that each one of the three cytokines synergizes with the other two to generate CIML NK cells with enhanced polyfunctionality.

Afterward, we studied the polyfunctionality of CIML NK cells exposed to ruxolitinib during the preactivation phase. Since ruxolitinib reduces the cytotoxic activity of CIML NK cells (Figure 2B), we presumed that the polyfunctionality of these cells could also be affected in response to the preactivation with the three cytokines. In order to address this issue, two different concentrations of ruxolitinib were used (0.1 and 1 µM), which correspond to the serum levels found in treated patients (52, 53). We observed that a concentration of 0.1 µM ruxolitinib slightly reduces the polyfunctionality of CIML NK cells, while the higher dose (1 µM) induces a significant reduction (Figure 3B). Of note, CIML NK cells exposed to both concentrations of ruxolitinib are significantly more polyfunctional than control non-preactivated NK cells, indicating that part of the effect mediated by the combination of IL-12, IL-15, and IL-18 is independent of Jak1/2 (Figure 3B).

### Phenotype of CIML NK Cells

**Figure 4:**
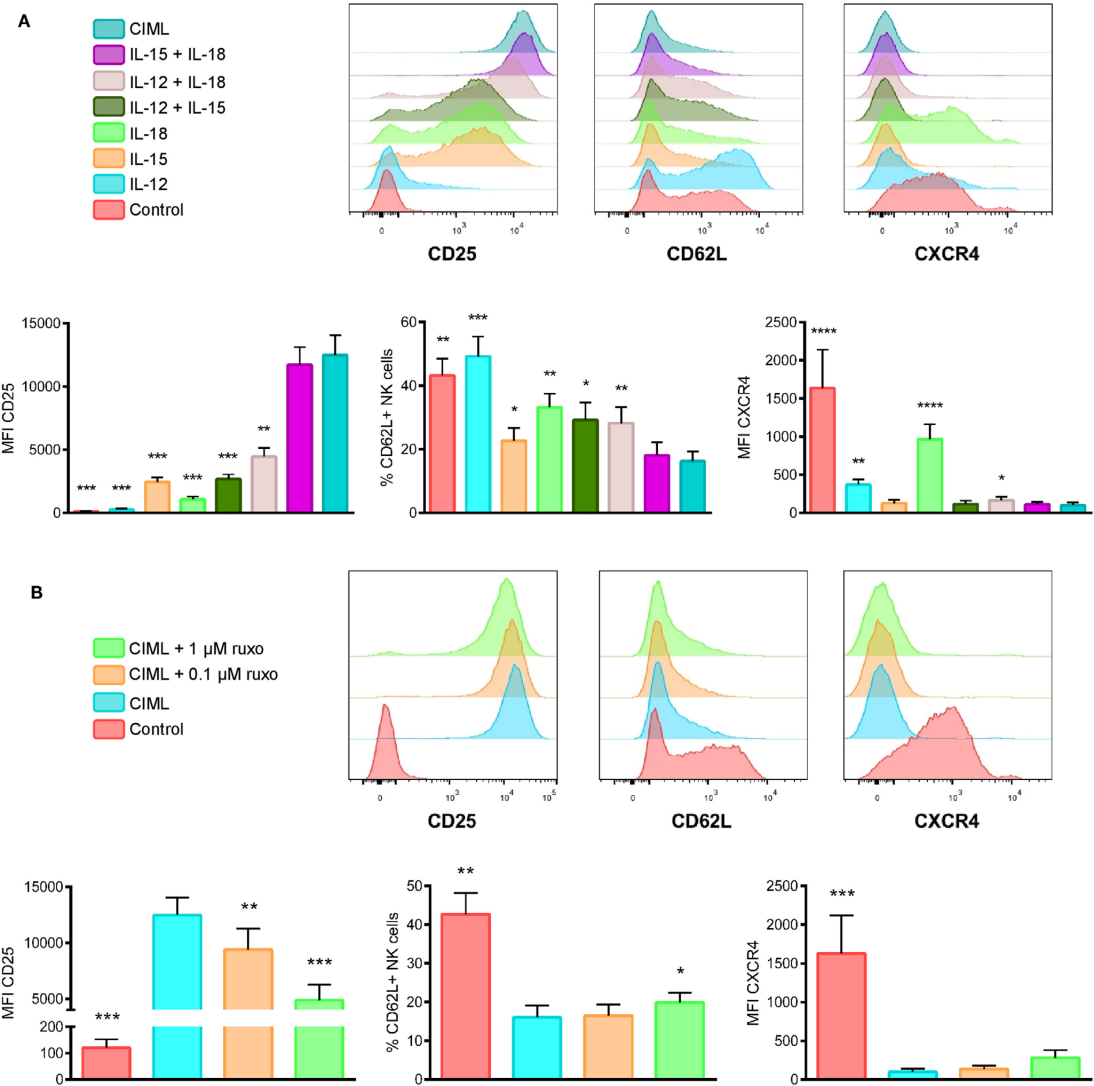
Phenotype of cytokine preactivated natural killer (NK) cells after the preactivation phase. **(A)** Representative experiment (top) and bar graphs (bottom) showing the expression of CD25, CD62L, and CXCR4 on control non-preactivated and cytokine preactivated NK cells (*n* = 8). **(B)** A representative experiment (top) and bar graphs (bottom) showing the expression of CD25, CD62L and CXCR4 on control non-preactivated and cytokine-induced memory-like (CIML) NK cells, and CIML NK cells preactivated in the presence of 0.1 and 1 µM ruxolitinib (*n* = 8). The bar graphs show the mean with SEM. Differences between CIML NK cells and the rest of conditions were established with RM one-way ANOVA with the Greenhouse-Geisser correction (for normally distributed data) or Friedman test (for non-normal distributed data). *p < 0.05, **p < 0.01, ***p < 0.001, ****p < 0.0001.

In order to be suitable and effective for adoptive cell therapy, CIML NK cells must be able to traffic to the tumor site. This trafficking is to a large extent regulated by several chemokine and other homing receptors. Thus, in the context of hematological malignancies, we analyzed the expression of CD62L and CXCR4, two cell surface receptors that are required for homing to lymph nodes and bone marrow, respectively (54, 55). Additionally, we also studied the expression of IL-2Rα (CD25), which conforms, along with the IL-2Rβγ, the high-affinity receptor IL-2Rαβγ that signals in response to picomolar concentrations of IL-2 (56). In line with the results obtained by other authors, ours showed that the expression of CD25 is specially increased when human NK cells are preactivated with IL-15 + IL-18 or IL-12 + IL-15 + IL-18 (26, 45, 57) (Figure 4A). Conversely, the expression of CXCR4 and the percentage of CD62L+ NK cells are reduced when they are preactivated. Interestingly, the expression of CXCR4 is specially reduced when NK cells are exposed to IL-15, either alone or in combination with other cytokines, indicating that IL-15 has the most relevant role in decreasing the cell surface expression of this chemokine receptor, while stimulation with IL-18 alone has little effect on CXCR4 expression (Figure 4A). Related to CD62L expression, we also observed that IL-15 is the most relevant cytokine for the decreased expression of this marker on CIML NK cells, while IL-12 alone has no effect (Figure 4A).

We also analyzed the effect of ruxolitinib on CIML NK cells phenotype. The expression of CXCR4 and the percentage of CD62L+ NK cells, which are decreased upon the stimulation with IL-12 + IL-15 + IL-18, are not severely affected by the inhibition of Jak1/2 during the preactivation phase. Contrariwise, we noted that the expression of CD25 tends to decrease in a ruxolitinib concentration-dependent manner; although the levels of this receptor are significantly higher in CIML NK cells generated in the presence of ruxolitinib than in control non-preactivated NK cells (Figure 4B).

### IL-2-Induced Proliferation of NK Cells Exposed to Different Combinations of Cytokines

**Figure 5:**
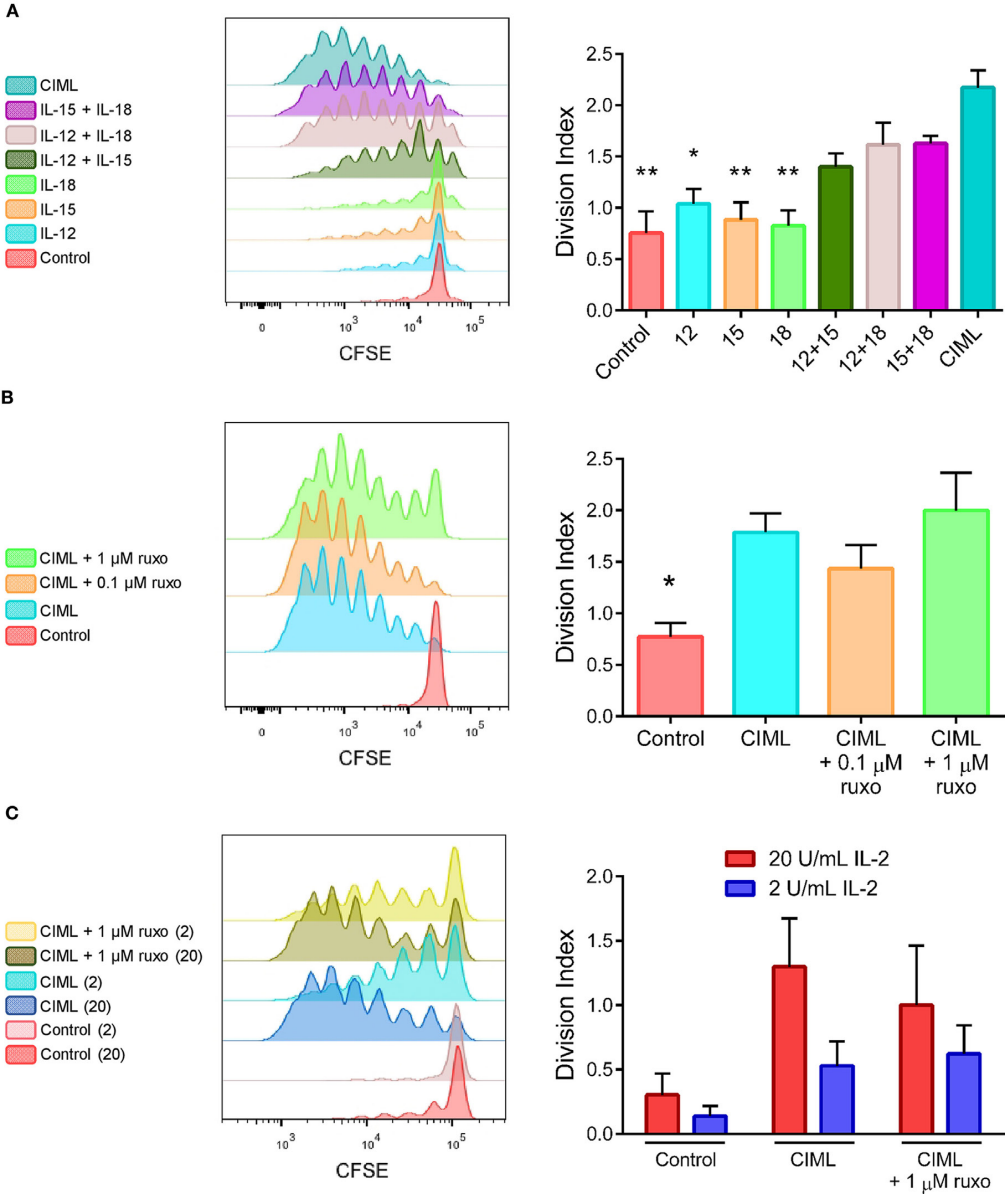
IL-2-induced proliferation of cytokine preactivated natural killer (NK) cells at the end of the expansion phase of 4 days. **(A)** Histograms of a representative experiment (left) and bar graphs showing the division index (DI) (right) of control non-preactivated and cytokine preactivated NK cells (*n* = 6). **(B)** A representative experiment (left) and bar graphs showing the DI (right) of control non-preactivated and cytokine-induced memory-like (CIML) NK cells, and CIML NK cells preactivated in the presence 0.1 and 1 µM ruxolitinib (*n* = 5). **(C)** A representative experiment (left) and bar graphs showing the DI (right) of control non-preactivated and CIML NK cells, preactivated in the presence and absence of 1 µM ruxolitinib, exposed to 2 or 20 U/mL IL-2 during the expansion phase (*n* = 5). The bar graphs show the mean with SEM. Differences between CIML NK cells and the rest of conditions were established with Friedman test. *p < 0.05, **p < 0.01.

IL-2 has been widely used in cancer immunotherapy for both stimulation of autologous NK cells and activation/expansion of donor NK cells (34, 36, 58). CIML NK cells (after 12–16 h of preactivation) were adoptively transferred to lymphodepleted patients with AML, and then, low doses of recombinant human IL-2 were administered to support memory-like NK cells expansion (27). We decided to study the proliferative potential of preactivated CIML NK cells in response to low concentrations of IL-2. In our experimental settings, after the preactivation phase, NK cells were washed and exposed to 20 U/mL of IL-2 for 4 days. We compared the proliferation of NK cells by calculating the division index (DI), defined as the average number of cell divisions that a cell in the original population has undergone (59). Our results showed that CIML NK cells proliferate significantly more than control non-preactivated NK cells and those exposed to just one cytokine alone (Figure 5A). Moreover, NK cells that were preactivated with the combination of two cytokines showed higher DI than those preactivated with just one cytokine, but lower DI than CIML NK cells, although is not statistically significant (Figure 5A).

We also studied whether the exposure to ruxolitinib during the preactivation phase could affect the posterior expansion of CIML NK cells in response to IL-2. Our findings revealed that Jak1/2 inhibition during the preactivation phase does not significantly reduce the proliferation of CIML NK cells (Figure 5B). Additionally, we cultured CIML NK cells with low or very low concentrations of IL-2 (20 and 2 U/mL, respectively) to test the effect of limiting amounts of this cytokine during the expansion phase. As expected, a very low dose of IL-2 induced a smaller proliferative response of CIML NK cells, which was not affected by the presence of ruxolitinib during the preactivation phase (Figure 5C).

### Ruxolinib-Treated CIML NK Cells Recover Their Polyfunctionality After the Expansion Phase

**Figure 6:**
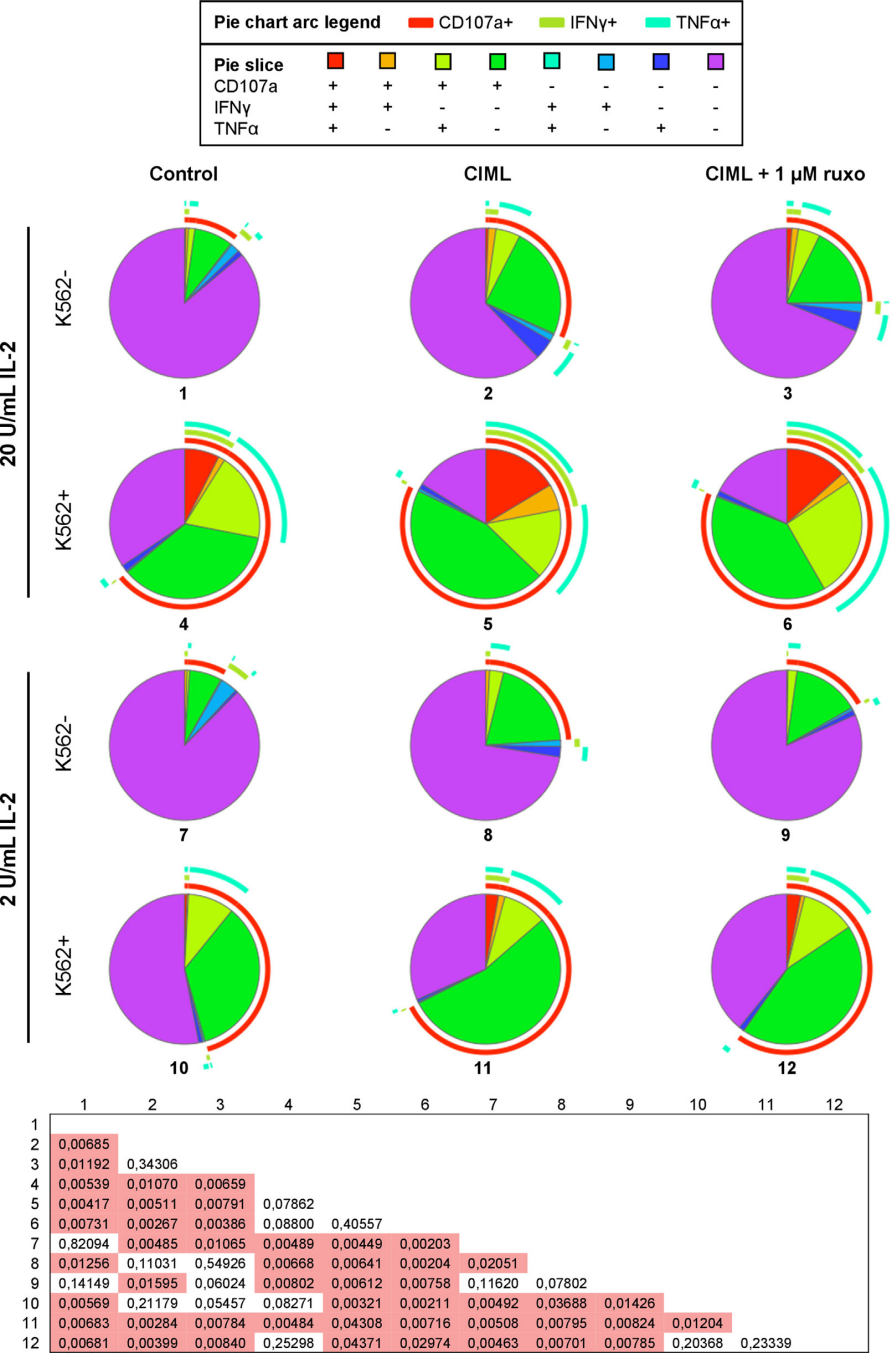
Polyfunctionality of cytokine-induced memory-like (CIML) natural killer (NK) cells after the expansion phase. Pie charts represent the percentages of control non-preactivated and CIML NK cells, preactivated in the presence and absence of 1 µM ruxolitinib, expressing CD107a, IFNγ, and/or TNFα. Cells were exposed to 2 U/mL (lower six pie charts) or 20 U/mL (upper six pie charts) of IL-2 during the expansion phase of 4 days, and then washed and incubated with or without K562 cells for 6 h (*n* = 6). Differences between pie charts were established with non-parametric permutation test. The *p* values are in the box below the pie charts. Significant differences are in the red cells.

The presence of ruxolitinib during the preactivation phase significantly reduces the polyfunctionality of preactivated CIML NK cells (Figure 3B). We studied whether this reduction is maintained or not after an expansion phase of 4 days, in the presence of IL-2. NK cells were preactivated with IL-12, IL-15, and IL-18, with or without ruxolitinib. Then, cells were washed and exposed to low or very low concentrations of IL-2 for 4 days. After the expansion phase, we analyzed their polyfunctional profile in the absence and after encountering K562 cancer cells. First, as expected, NK cells cocultured with K562 target cells exhibited a significantly higher polyfunctional profile. Furthermore, CIML NK cells were more polyfunctional than control non-preactivated NK cells when exposed to IL-2 for 4 days. Importantly, there are no statistically significant differences between CIML NK cells that were preactivated in the presence and absence of ruxolitinib (Figure 6), indicating that their polyfunctionality, both without and after encountering K562 tumor cells, is not substantially affected by the exposure to Jak1/2 inhibitor during the preactivation phase. These results indicate that the effect of ruxolitinib on the polyfunctionality of CIML NK cells during the preactivation phase is reversible, so that after the expansion with low and very low doses of IL-2 the consequences of exposure to this drug disappear. This can be explained by the fact that CIML NK cells generated in the presence of ruxolitinib still express enough levels of CD25 that allow them to respond to low doses of IL-2 (Figure 4B).

### Ruxolitinib Has No Severe Impact on the Expression of CD62L and CXCR4 Homing Receptors in Expanded CIML NK Cells

**Figure 7:**
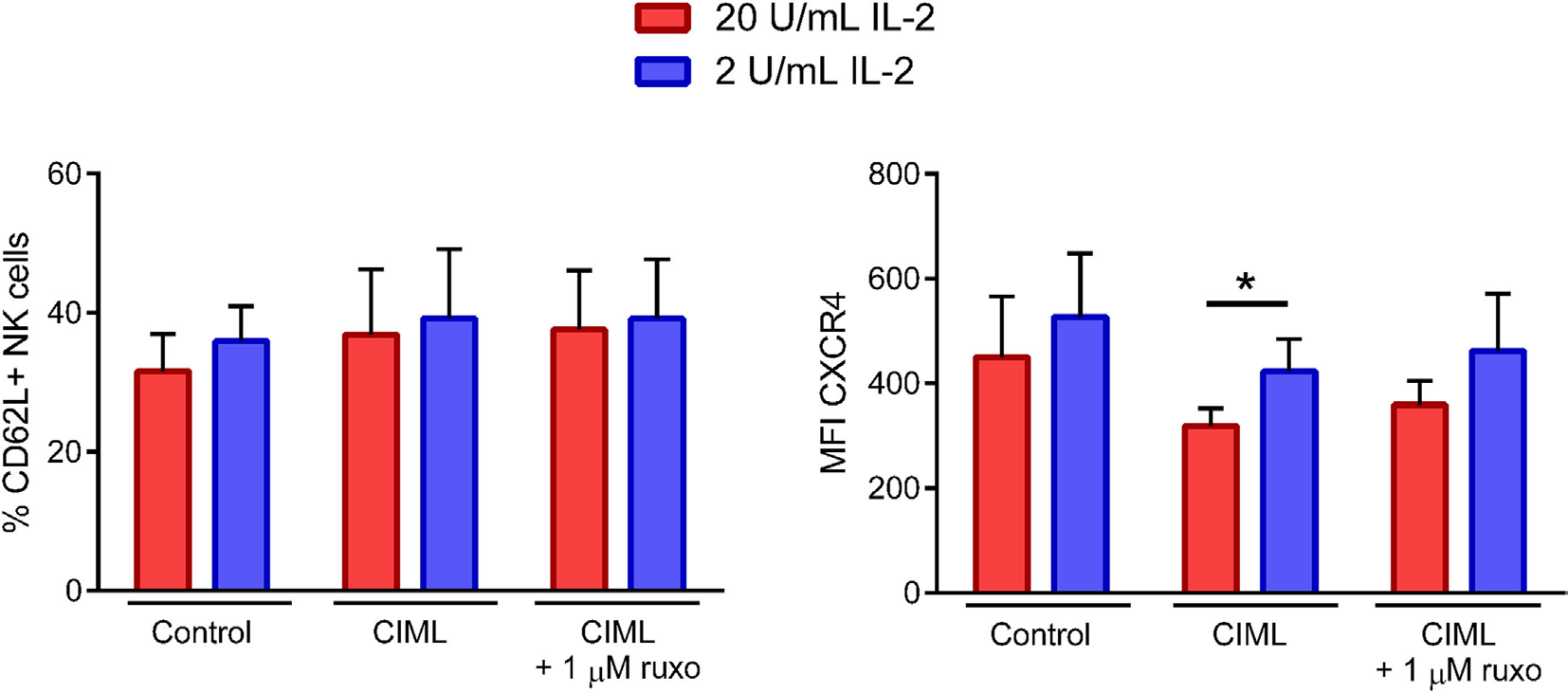
CD62L and CXCR4 expression of cytokine-induced memory-like (CIML) natural killer (NK) cells after the expansion phase. Bar graphs showing the percentage of CD62L+ NK cells and the expression (MFI, median fluorescence intensity) of CXCR4 in control non-preactivated and CIML NK cells, preactivated in the presence and absence of 1 µM ruxolitinib, exposed to 2 or 20 U/mL IL-2 during the expansion phase (*n* = 6). Bar graphs show the mean with SEM. Statistical differences were established with Wilcoxon matched-pairs signed rank test. *p < 0.05.

Finally, we analyzed the expression of CD62L and CXCR4 homing receptors after the expansion phase. Our results showed that the percentage of CD62L+ NK cells (Figure 7, left panel) was similar between the three tested conditions (i.e., control non-preactivated and CIML NK cells exposed or not to ruxolitinib), while the expression of CXCR4 (Figure 7, right panel) was reduced when NK cells were preactivated, both in the presence and absence of the Jak1/2 inhibitor. Notably, although the concentration of IL-2 during the expansion phase had little or no effect on the percentage of CD62L+ cells, the expression of CXCR4 was higher when cells were exposed to lower doses of IL-2, somehow suggesting that cell activation induces a downregulation of CXCR4 (Figure 7). Our findings revealed that the expression of CXCR4 after the expansion phase was partially affected by the concentration of IL-2, but not by the exposure of NK cells to ruxolitinib during the preactivation phase.

## Discussion

Natural killer cells play a pivotal role in the immunosurveillance of malignant cells (1, 2, 33, 60–62). They have become a promising tool in cancer immunotherapy with an increasing number of basic and translational studies focused on them, including those about CIML NK cells (2, 27–29, 32, 38, 45, 61, 63–66). Several studies have shed light on the *in vivo* antitumor properties of CIML NK cells and their potential in the treatment of AML (27, 45). However, a more extensive characterization of their effector functions, proliferative capacity and phenotype, of these cytokine preactivated NK cells, it is required in order to design the best immunotherapy. In our study, we sought to describe the synergistic effect that IL-12, IL-15, and IL-18 combinations have on the cytotoxicity, polyfunctionality, expression of homing receptors, and IL-2-induced proliferation of human NK cells *in vitro*. Moreover, we have tested the effect of ruxolitinib, a Jak1/2 inhibitor, in the generation of CIML NK cells, in an attempt to better understand the roles of IL-12R- and IL-15R-mediated signals during the preactivation phase.

Our data provide evidence of a major contribution of IL-15 to CIML NK cell-mediated cytotoxicity against K562 cancer cells. In line with our results, other authors have previously demonstrated that stimulation with IL-15 enhanced the cytotoxicity of NK cells against K562 targets, primary acute leukemic blasts, and rhabdomyosarcoma cell lines (67–70). The enhanced responses of IL-15-primed NK cells are the outcome of several factors, including the upregulation of several NK cell activating receptors and increased production of cytokines and cytotoxic proteins (68, 70–72).

Nevertheless, our functional analysis of CIML NK cells was not limited to their cytotoxic potential. We extended our study of these cells in an attempt to thoroughly investigate more effector functions. Our data revealed that cytokine preactivation reduces the percentage of quadruple negative (CD107a− IFNγ− TNFα− CCL3−) NK cells, and that this percentage is even lower as more cytokines are used during the preactivation phase. In this line, the combination of IL-12 + IL-15 + IL-18 (i.e., CIML NK cells) shows the lower percentage of cells negative for the tested functions, supporting the idea that the synergistic effect of these three cytokines is required to endow NK cells the best polyfunctional profile. Each one of the three cytokines contributes to the final phenotypical and functional features that CIML NK cells exhibit. For example, in this context, it has been previously reported that IL-12-mediated signals are essential for the generation of human NK cells with enhanced effector functions after restimulation (30, 73). Chaix et al. have described the cooperation between IL-12 and IL-18, where signaling through IL-18R/MyD88/IRAK4 pathway was required for IFNγ production by NK cells in response to IL-12 (74). Accordingly, other authors have proposed IL-18 as the key cytokine that primes NK cells to respond to other cytokines (57). IL-15 can be also used to prime NK cells, although the improved functionality persists no longer than 72 h after stimulation (70), in comparison with CIML NK cells that show sustained antitumor activity and can be detected up to 3 months after adoptive transfer in mice (27, 29, 45).

The small number of donors in our study could be a limitation since there are several factors that we have not considered. For instance, HCMV infection status has a significant influence on cytokine responsiveness of NK cells. Goodier et al. reported that the production of IFNγ and the expression of CD107a and CD25 by cytokine-stimulated NK cells were different between HCMV+ and HCMV− individuals (75). In addition, NK cell responses are also strongly influenced by KIR-HLA interactions. Although we have not analyzed the KIR repertoire, several studies have revealed the impact of cytokines on KIR cell surface expression. Ewen et al. have found that the activation of NK cells with IL-12/15/18 led to a decreased expression of KIR2DL1, KIR2DL2/L3, and KIR3DL1. They found that this downregulation was not detectable before 36 h of cytokine-stimulation and that the most pronounced downregulation was observed around 48–60 h after stimulation (63). Other authors have also showed the capacity of IL-2 to modulate the KIR repertoire (76, 77). However, Romee et al. reported that allogeneic adoptive transferred CIML NK cells showed a similar production of IFNγ in both KIR-mismatched and -matched situations against primary AML blasts (27). On the other hand, Horowitz et al. have shown that a dimorphism at position −21 of the HLA-B leader sequence has a great impact on the NK cell IFNγ response upon IL-12/15 stimulation (78). In the future, it will be of great interest to study KIR–HLA interactions, HCMV status, and other factors in our experimental settings.

Cytokine pre-stimulation induces phenotypic changes that may influence, to a greater or lesser extent, trafficking and proliferation of NK cells. We found differential expression of CXCR4, CD62L, and CD25 markers following cytokine stimulation, which depend on the interleukin combination we used to preactivate the NK cells. Our results show that CXCR4 is drastically downregulated when IL-15 is present in the cytokine cocktail, or a combination of two or three cytokines is used during the preactivation phase. However, these differences in the expression of CXCR4 between control non-preactivated and CIML NK cells are not maintained for a long time. As shown, after the expansion phase in the presence of 20 U/mL of IL-2, the expression levels of CXCR4 is reduced, both in control non-preactivated and CIML NK cells (Figure 7). Likewise, it has been previously reported that NK cells stimulated with high doses (1,000 U/mL) of IL-2 (whose receptor shares the β and γ subunits with IL-15R) for 5 days decreased CXCR4 expression levels and homing/accumulation to the bone marrow (54). Since CIML NK cells are being used for the treatment of hematological malignancies (clinical trials NCT01898793, NCT03068819, and NCT02782546), it would be preferable that they express higher levels of CXCR4 in order to improve the trafficking to the bone marrow. As we have demonstrated, expanding CIML NK cells with very low doses of IL-2 (2 U/mL) resulted in a higher expression of this marker in comparison to those cells exposed to 20 U/mL, thus highlighting the impact of the dose of IL-2 given during the expansion phase. Nevertheless, Romee et al. have shown that, after 7 days, adoptively transferred human CIML NK cells are able to localize in key hematopoietic tissues (i.e., bone marrow, spleen, and blood) in similar numbers compared to control non-preactivated NK cells in NSG mice, even in the presence of IL-2. Furthermore, they have reported that at day 8 post-infusion, CIML NK cells are present at large percentages in the bone marrow of AML patients who were also treated with IL-2 (27). Thus, although the reduced expression of CXCR4 seems not to be a limiting factor for homing CIML NK cells to the bone marrow, it is not clear if the administration of even lower doses of IL-2 may increase the trafficking of CIML NK cells. Additional *in vivo* studies should be performed to clarify this issue. It would be very interesting to comprehensively study CXCR4 regulation. For example, to investigate whether cytokine stimulation has some effects on the proteins that regulate its surface expression and endocytosis (i.e., filamin A and β-arrestin-1) (79). Moreover, several studies have shown that CXCR4 expression can be upregulated with glucocorticoids (80, 81), TGF-β1 (82, 83), or the BCR-ABL tyrosine kinase inhibitors imatinib and nilotinib (84). Hence, preactivation of NK cells with IL-12, IL-15, and IL-18 should be tested in the presence of these drugs.

Our results also show that CD62L is downregulated on CIML NK cells after cytokine stimulation, which is in accordance with the results obtained by others (29, 85, 86). In contrast, Romee et al. have reported that the expression of CD62L is upregulated on CIML NK cells after a resting period of 7 days (27), while in our experiments, NK cells were being stimulated with IL-2. The main responsibility of CD62L downregulation is A Disintegrin and Metalloprotease 17 (ADAM17) (86). Thus, the use of specific inhibitors of ADAM17 during the preactivation phase could inhibit CD62L downregulation, as it has been recently demonstrated on NK cells stimulated with IL-12 + IL-18 (86). In a broader context, matrix metalloproteases inhibitors are able to impede CD16 and CD62L downregulation following NK cell activation (50, 86–88). Therefore, if the objective is to increase the expression of CD62L, it would be interesting to explore the generation of CIML NK cells while inhibiting matrix metalloproteases. Furthermore, matrix metalloprotease inhibition promotes NK cell polyfunctionality in antibody-based antitumor immunotherapy (50, 86).

The expression of CD25 is outstandingly upregulated when NK cells are stimulated for 16–18 h with IL-15 + IL-18 or IL-12 + IL-15 + IL-18, which is in agreement with the results obtained from other authors (26, 57). Moreover, Leong et al. have described that IL-2 stimulates the proliferation of NK cells preactivated with IL-15, IL-12 + IL-18, or IL-15 + IL-18 and have linked the expression of CD25 to IL-2-induced proliferation in those cells (26). We have investigated the proliferation of NK cells preactivated with all the combinations of IL-12, IL-15, and IL-18, and found that CIML NK cells have the highest DI. However, the combination of two cytokines also has a significant impact on NK cell proliferation.

In addition to the contribution of each cytokine to NK cell phenotype and effector functions, we have also examined the effect of Jak1/2 inhibition during the preactivation phase. Schönberg et al. reported that ruxolitinib impairs K562 killing of IL-2 preactivated NK cells (89). Our protocol is somehow different from the one that Schönberg et al. have followed. In our experiments, NK cells were exposed simultaneously to ruxolitinib and cytokines, and thoroughly washed after the preactivation phase to remove them from the medium. Nevertheless, we have also shown that ruxolitinib has a noticeable effect on the functionality of CIML NK cells just after the preactivation phase, reducing their cytotoxic activity against K562 targets and their polyfunctionality in a dose-dependent manner. It has been described that Jak1/2 inhibition prevented IL-2 induced upregulation of granzyme B, NKp46, NKG2D, and CD69 (89). Also, Jak1/2 inhibition reduced degranulation and IFNγ production of NK cells stimulated with monocyte-derived dendritic cells (90). These findings suggest that ruxolitinib diminished NK cell functionality, which are in agreement with the reduced cytotoxicity against K562 target cells of ruxolitinib-exposed control non-preactivated and CIML NK cells. However, the effects of ruxolitinib on degranulation and specific killing are reversible (89). Accordingly, we have measured the polyfunctionality after 4 days of expansion phase and found that CIML NK cells that were exposed to the highest dose of ruxolitinib (1 µM) showed no differences in their polyfunctional profile compared to non ruxolitinib treated CIML NK cells, reinforcing the finding that the effects of ruxolitinib are reversible.

Jak1/2 inhibition does not prevent the downregulation of homing receptors CD62L and CXCR4 in CIML NK cells. Furthermore, the high CD25 expression on CIML NK cells is significantly reduced when they are preactivated in the presence of 1 µM ruxolitinib. To evaluate its impact, we analyzed IL-2-induced proliferation and found no significant differences between CIML NK cells exposed or not to ruxolitinib. Therefore, we conclude that, even though the expression of CD25 is reduced, it is sufficient to promote the proliferation of CIML NK cells exposed to low doses of IL-2. Indeed, the concentration of IL-2 during the expansion phase, rather than Jak1/2 inhibition, could be a limiting factor for NK cell proliferation, as we have demonstrated. Altogether, our results suggest that the exposure of CIML NK cells to ruxolitinib during the preactivation phase could mitigate the initial impact that these highly activated NK cells, producing very high amounts of cytokines, may have on the patient right after the cell infusion.

## Ethics Statement

All subjects provided written and signed informed consent in accordance with the Declaration of Helsinki. The protocol was approved by the Basque Ethics Committee for Clinical Research.

## Author Contributions

IT designed and performed experiments, analyzed and interpreted the data, designed the figures, and wrote the manuscript. IM wrote the manuscript and analyzed data. IO, AG and JG performed experiments, analyzed and interpreted the data. AO and JV participated in the analysis and interpretation of the data. OZ performed experiments, participated in the design of the study and interpreted the data. FB conceived and designed the study, interpreted the data, and wrote the manuscript. All the authors critically reviewed, edited and approved the final manuscript.

## Conflict of Interest Statement

The authors declare that the research was conducted in the absence of any commercial or financial relationships that could be construed as a potential conflict of interest.
