# Supplementary material for: Implication of Interleukin-12/15/18 and Ruxolitinib in the Phenotype, Proliferation, and Polyfunctionality of Human Cytokine-Preactivated Natural Killer Cells
Source: Front Immunol. 2018 Apr 16;9:737. doi: 10.3389/fimmu.2018.00737 (PMC5911648; doi:10.3389/fimmu.2018.00737)
Supplement: Supplementary file 1 [file Image_1.PDF]

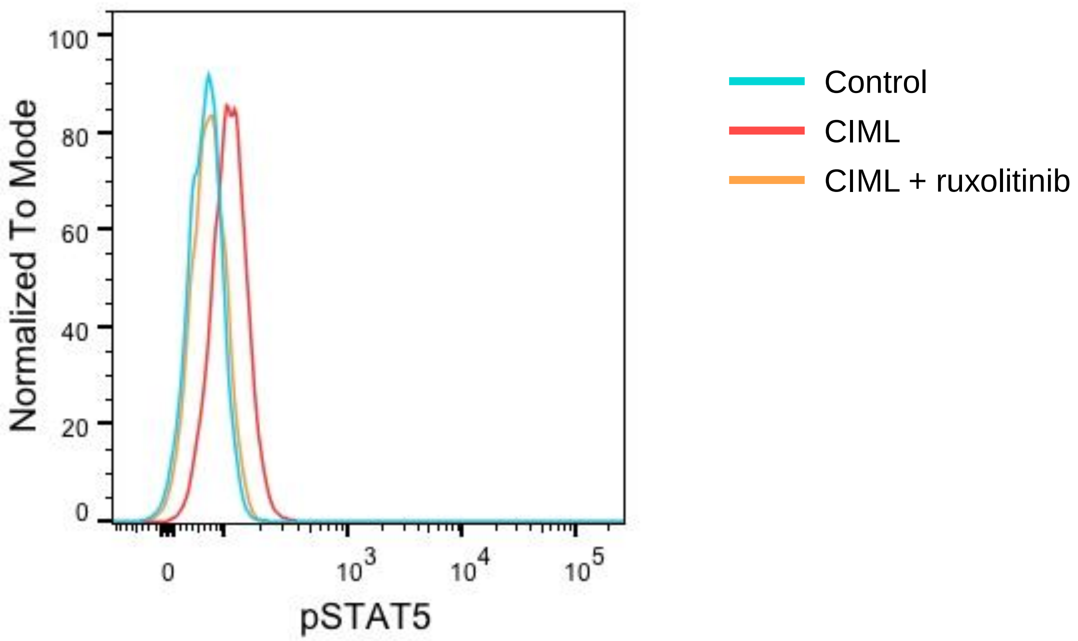

Figure S1. Ruxolitinib impairs the phosphorylation of STAT5. The histograms represent the levels of phosphorylated STAT5 (pSTAT5) of control non-preactivated and CIML NK cells stimulated, in the presence and absence of 0.1 µM ruxolitinib, with IL-12 (10 ng/mL), IL-15 (10 ng/mL) and IL-18 (50 ng/mL).
